# Score-Guided Structural Equation Model Trees

**DOI:** 10.3389/fpsyg.2020.564403

**Published:** 2021-01-28

**Authors:** Manuel Arnold, Manuel C. Voelkle, Andreas M. Brandmaier

**Affiliations:** ^1^Psychological Research Methods, Department of Psychology, Humboldt-Universität zu Berlin, Berlin, Germany; ^2^Max Planck UCL Centre for Computational Psychiatry and Ageing Research, Berlin, Germany; ^3^Center for Lifespan Psychology, Max Planck Institute for Human Development, Berlin, Germany

**Keywords:** exploratory data analysis, heterogeneity, model-based recursive partitioning, parameter stability, structural change tests, structural equation modeling

## Abstract

Structural equation model (SEM) trees are data-driven tools for finding variables that predict group differences in SEM parameters. SEM trees build upon the decision tree paradigm by growing tree structures that divide a data set recursively into homogeneous subsets. In past research, SEM trees have been estimated predominantly with the R package semtree. The original algorithm in the semtree package selects split variables among covariates by calculating a likelihood ratio for each possible split of each covariate. Obtaining these likelihood ratios is computationally demanding. As a remedy, we propose to guide the construction of SEM trees by a family of score-based tests that have recently been popularized in psychometrics (Merkle and Zeileis, 2013; Merkle et al., 2014). These score-based tests monitor fluctuations in case-wise derivatives of the likelihood function to detect parameter differences between groups. Compared to the likelihood-ratio approach, score-based tests are computationally efficient because they do not require refitting the model for every possible split. In this paper, we introduce score-guided SEM trees, implement them in semtree, and evaluate their performance by means of a Monte Carlo simulation.

## Introduction

Structural equation models (SEMs; Bollen, 1989; Kline, 2016) are a widely applied technique in social and psychological research to model the relationships between multiple variables. SEMs are especially useful when some of the variables under investigation are latent (not directly observable) or contain measurement errors. Various statistical procedures such as factor analysis, ANOVA, linear regression, mediation models, growth curve models, and dynamic panel models can be specified within the SEM framework.

A major challenge that complicates the specification and interpretation of SEMs are potential differences between subgroups of the sample. Group differences can pertain to various aspects of a SEM. For instance, in a latent growth curve model, we may find differences in how people change over time, or in a factor analysis model, the factor structure may vary across groups. By neglecting such instances of sample heterogeneity, SEM parameter estimates may not represent any individual in the sample, and researchers risk drawing incorrect conclusions from their data (e.g., Kievit et al., 2013). This makes identifying group differences in SEM parameters an important task.

One popular strategy is to detect heterogeneity in SEMs with the help of covariates. Multi-group structural equation models (MGSEMs; Sörbom, 1974) allow estimating different parameter values for the levels of a grouping variable, such as males and females or treated versus non-treated. By comparing the fit of a single-group SEM to the fit of a MGSEM, equality constraints on parameters across groups can be tested with the likelihood-ratio test. Multi-group structural equation modeling excels as a confirmatory tool to test a limited number of hypotheses about group differences. As part of exploratory data analysis, however, the method can often become tedious in large data sets. With many potentially important grouping variables, many MGSEMs need to be specified and estimated. Moreover, since MGSEMs require discrete grouping variables, numeric and ordinal covariates such as age or socioeconomic status need to be discretized, which often leads to a loss of information (but see Hildebrandt et al., 2016).

SEM trees, as first presented by Brandmaier et al. (2013b), can be seen as an extension of MGSEMs for exploring parameter heterogeneity in SEMs. SEM trees are a data-driven approach that automatically searches through all available covariates to identify partitions of the full sample that differ with respect to SEM parameter estimates. SEM trees build upon the model-based recursive partitioning paradigm (for an overview, see Zeileis et al., 2008; Strobl et al., 2009). One key feature of SEM trees is their interpretability: SEM trees provide a graphical representation of how covariates and covariate interactions predict non-linear differences in SEM parameters. The building blocks of SEM trees are called nodes, each containing a SEM fitted to a distinct subsample. The SEM tree algorithm forms a binary tree structure by hierarchically splitting these nodes. Each node of the SEM tree has either two successors (daughter nodes) and is called an *inner node* or no successors and is called a *leaf* (or terminal node). The first node of the tree is called the *root* and has no parent nodes. The inner nodes of the tree represent split decisions. Each split decision involves a covariate (e.g., age of the observed individuals) and a cut point in the covariate (e.g., divide the sample into individuals younger and older than 45 years). A leaf of a tree contains a partition of the sample that is best described with a set of SEM parameters. All leaves taken together exhaustively partition the original sample and can be thought of as a MGSEM with potentially many groups. An important difference to conventional multi-group structural equation modeling is that the group membership in a SEM tree is not pre-specified but learned from the data.

There are currently two software packages for the statistical programming language R that allow fitting SEM trees. One is the semtree package (Brandmaier et al., 2013b) that has been widely applied in the literature (Brandmaier et al., 2013a, 2016, 2017, 2018; Jacobucci et al., 2017; Usami et al., 2017, 2019; de Mooij et al., 2018; Ammerman et al., 2019; Serang et al., 2020; Simpson-Kent et al., 2020). The other software implementation is the partykit package (Hothorn and Zeileis, 2015). Unlike semtree, partykit is not limited to a specific model class such as SEMs but provides the infrastructure for general recursive partitioning across various model classes. Among other features, partykit provides the generic MOB algorithm for model-based recursive partitioning that has been used to study heterogeneity in M-estimators (Zeileis et al., 2008), Bradley-Terry models (Strobl et al., 2011), Rasch models (Strobl et al., 2015; Komboz et al., 2018), multinomial processing trees (Wickelmaier and Zeileis, 2018), generalized linear mixed-effects models (Fokkema et al., 2018), network models (Jones et al., 2020), and circular regression models (Lang et al., 2020). Moreover, MOB is also used in more specialized recursive partitioning packages such as psychotree (Zeileis et al., 2020). Recently, Zeileis (2020) demonstrated on his blog how MOB can be coupled with the SEM software lavaan (Rosseel, 2012) to estimate SEM trees. Outside of the R ecosystem, SEM trees have also been fitted in M*plus* (Serang et al., 2020).

SEM trees are estimated by recursively selecting the covariate that best partitions the sample into different subgroups. Thus, the evaluation of potential splits is the central aspect of the algorithm. The semtree package uses a procedure that transforms all non-dichotomous covariates (that is, covariates with more than two values) into a set of dichotomous split candidates. Then, the tree growing algorithm computes the likelihood ratio between a single SEM (fitted on the complete sample of the current node) and MGSEMs (representing the model after the split) for every split candidate and selects the candidate associated with the largest likelihood ratio. The number of MGSEMs needed to calculate these likelihood ratios is directly related to the number of possible splits of the covariate. For instance, evaluating a numeric covariate such as age with many different values will require more MGSEMs to be estimated than evaluating a discrete covariate such as handedness. The reliance of the semtree package on likelihood ratios has the apparent drawback that the computational burden becomes large to excessive if there are many covariates and the covariates have many unique values. Another problem of the current semtree package is that the standard approach to split evaluation (called *naïve* selection approach in semtree) is biased by favoring the selection of covariates with many unique values over covariates with few unique values (Brandmaier et al., 2013b). The semtree package offers a correction procedure (*fair* selection approach) for this selection bias (also known as attribute selection error; Jensen and Cohen, 2000). However, this correction procedure is heuristic and comes at the price of decreased statistical power to detect group differences. To solve this problem, we suggest to use a well-known method for likelihood-ratio-guided covariate selection that does not suffer from a selection bias while retaining full statistical power. We implemented this method into the semtree package.

In contrast to the semtree package, model-based recursive partitioning in the partykit package uses so-called score-based or structural change tests (e.g., Zeileis and Hornik, 2007) for assessing whether the values of one or more parameters depend on a covariate. Score-based tests are obtained by cumulating the case-wise gradients of the log-likelihood function evaluated at the parameter estimates. Unlike the likelihood-ratio test, score-based tests do not require the estimation of group-specific models for the evaluation of each split. This property leads to two advantages that make score-based tests highly attractive for model-based recursive partitioning. First, they are computationally efficient, as only the pre-split model needs to be estimated once. Second, when subgroups become small, fitting multi-group models to obtain likelihood ratios may become unstable. We propose using the advantages of score-based tests and added SEM trees guided by score-based tests to the semtree package. Our implementation of score-guided trees differs in some points from the generic MOB algorithm from the partykit package. MOB uses score-based tests to select a covariate, and it locates the optimal cut point in this covariate by comparing likelihood ratios. In contrast, our semtree implementation uses a score-based cut point localization, which is computationally more efficient. Moreover, MOB is currently limited to a single score-based test statistic, whereas semtree offers a broader selection of different test statistics that recently became popular in the exploration of measurement invariance in SEMs (Merkle and Zeileis, 2013; Merkle et al., 2014; Wang et al., 2014, 2018).

The present study assesses a wide range of variable selection techniques in a Monte Carlo simulation study using the semtree package. We implemented an optimal likelihood-ratio-based method to improve the statistical properties of the likelihood-ratio-based split selection in semtree and added a family of score-based tests as a computationally efficient alternative. We evaluated the performance of these new methods next to the classical *naïve* and *fair* methods. Moreover, we explored two techniques offered by semtree that allow testing specific hypotheses and incorporating *a priori* knowledge about group differences. The remainder of this manuscript is organized as follows: first, we reiterate the basic principles of SEM trees. Second, the existing likelihood-ratio-based implementation is outlined in detail and complemented with an unbiased method for selecting covariates. Third, we recapitulate a family of score-based tests and show how they can be used to guide the split decision of SEM trees. Fourth, the simulation setup and results are shown. The study concludes with a discussion of the simulation results and recommendations for future research.

## Introductory Example

In the following, we illustrate the rationale behind SEM trees with an instructive example. Readers familiar with SEM trees may skip this section.

Let us assume a researcher estimated a confirmatory factor analysis (CFA; Brown, 2015) model that explains the scores of three ability tests of 600 male and female test takers of different ages with a single common latent factor and test-specific error terms. The data were collected at two different testing facilities. The researcher wonders if the parameter values of her CFA model differ with respect to the sites, the test takers’ age, and gender. She investigates this question with the help of a SEM tree.

The data for this fictional example were simulated such that the factor loading of the first ability test for individuals older than 45 years was smaller (0.6) than for younger individuals (0.8). This represents a violation of measurement invariance; that is, differences among individuals’ responses to an item are not only due to differences in the latent factor but also due to the item functioning differently across groups and being measured with different precision. Further, we lowered all factor loadings of older individuals tested at the second site by 0.1, imposing another form of violation of metric invariance. The covariate gender had no impact on the parameters of the CFA model and served as a noise variable.

**FIGURE 1:**
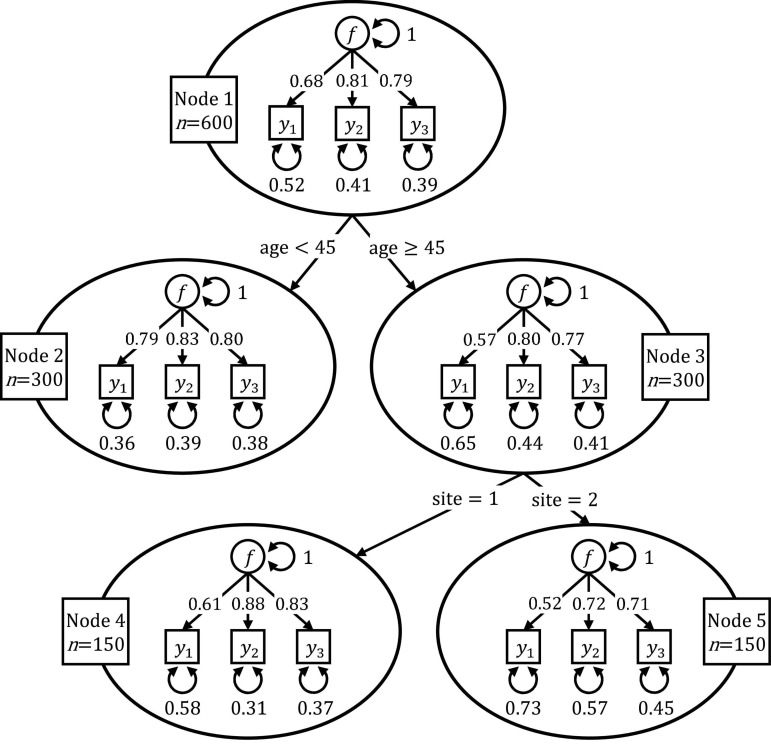
Illustrative example of a SEM tree. The SEM tree recursively partitioned a CFA model with respect to individuals’ age and study site.

Figure 1 shows the resulting SEM tree for the simulated data set. The SEM tree consists of 5 nodes depicted as ovals, each of them containing a CFA model. Node 1 is the root node of the SEM tree and contains the CFA model fitted on the full data set with *N* = 600 individuals. In this illustrative example, the SEM tree algorithm concluded that the fit of the model in the root node could be improved most by splitting the data into a group of 300 individuals younger than 45 years (Node 2) and a group of 300 individuals older than 45 years (Node 3). Node 2 and 3 are said to be the daughters of Node 1. After splitting the sample associated with Node 1, the algorithm proceeds recursively with Node 2 and 3. Whereas the fit of the model for younger individuals (Node 2) could not be improved any further, the SEM tree algorithm split the group of older individuals (Node 3) into two subgroups with 150 older individuals tested at site 1 (Node 4) and 150 older individuals tested at site 2 (Node 5). After this split, the SEM tree algorithm terminated as no further split would significantly improve any of the submodels’ fit. Nodes 2, 4, and 5 are the leaves of the SEM tree, and individuals within these nodes were found to be homogeneous with respect to the covariates. As expected, the SEM tree algorithm did not select the covariate gender for splitting because this covariate was not associated with any group differences in the simulated data set.

It is important to note that the structure of the SEM tree shown in Figure 1 is not specified *a priori* but learned top-down in an exploratory way. The algorithm only requires a pre-specified template SEM (in the example, the CFA model) and a data set including covariates that serve as split candidates to identify homogeneous groups. The selection of covariates and the identification of optimal cut points are then learned from the data. Throughout the tree, the structure of the template SEM remains the same, and only the values of the parameter estimates change as the model is fitted recursively on different subsamples.

## Structural Equation Model Trees

The generical SEM tree algorithm can be described in four steps:

1. Specify a template SEM.
2. Fit the template SEM to all observations in the current node.
3. Assess whether the SEM parameter estimates are constant or vary with respect to the covariate.
4. Choose the covariate that is associated with the largest group differences. If the group difference exceeds a threshold, split the node into two daughter nodes, and repeat the procedure with Step 2 for both daughter nodes. Otherwise terminate.

Likelihood-ratio-guided and score-guided SEM trees differ in how Step 3 of the general SEM tree algorithm is implemented. In other words, the procedures use different approaches to evaluate heterogeneity and to search for optimal split points in covariates. The following section outlines Steps 1–4 for likelihood-ratio-guided SEM trees before introducing score-guided SEM trees afterward.

### Step 1: Specification of the Template Model

The starting point for growing a SEM tree is the specification of a template SEM. A template model reflects hypotheses about the data by specifying relations among observed variables and latent constructs and is determined by the research question. The template model is fitted on all subsamples associated with the nodes of the SEM tree. It is important to note that the structure of the template model stays the same in the entire SEM tree (but see Brandmaier et al., 2013a for trees with multiple models). Hence, parameters fixed to a constant (e.g., zero or one) in the template model are fixed to the same constant in all submodels of the tree. Only parameters freely estimated in the template model are allowed to differ across groups and contribute to the assessments of splits.

Fixing many parameters of the template model to constants can hinder the SEM tree algorithm from identifying group differences. Usually, some parameters are fixed to ensure the identification of the SEM. In some model classes, additional constraints are specified to model specific relationships or trajectories. For instance, in latent growth curve models (see McArdle, 2012 for an overview), the factor loadings of a latent random slope variable are often fixed to model a specific growth pattern such as linear or quadratic growth. By fixing these loadings, a SEM tree will not be able to estimate different growth patterns between groups and, as a result, may overlook heterogeneity. In this case, estimating the factor loadings as free parameters may improve the SEM tree’s flexibility to adapt to subgroup-specific trajectories.

By default, SEM trees estimate all non-fixed parameters freely in each submodel, and every parameter contributes to the evaluation of split candidates. This behavior is suboptimal if there is a clear set of target parameters that are of interest to investigate a given theory. As a solution, the semtree package offers the option to specify a set of so-called focus parameters. By declaring focus parameters, the SEM tree will only consider heterogeneity in these parameters when assessing split candidates. Thus, focus parameters are useful for testing parameter-specific hypotheses about group differences. For instance, if one wants to test measurement invariance, one could specify the measurement model’s parameters as focus parameters and disregard heterogeneity in the structural model. Besides focus parameters, the semtree package allows constraining specific parameters to be equal across all submodels of the tree. This is done by estimating these parameters in the full sample once and using the resulting values throughout the tree. Such equality constraints allow incorporating prior knowledge about homogeneous parameters and can increase the power to detect heterogeneity in the remaining parameters. We will later demonstrate the use of focus parameters and equality constraints in two short simulation studies.

### Step 2: Model Estimation

Various estimation techniques for SEMs have been discussed. In principle, SEM trees can operate with any estimation method that provides a fit statistic and are not necessarily limited to a multivariate normal distribution. At present, however, only maximum likelihood estimation for multivariate normal data is implemented in the semtree package. Therefore, semtree is currently less suited for investigating models fitted on non-normal data such as SEMs with categorical outcomes. In the following, we will focus on maximum likelihood estimation for multivariate normal data.

SEMs are usually specified by expressing the structure of a mean vector and a covariance matrix as a function of a *q*-variate vector θ with model parameters. These parameters are estimated by minimizing a fitting function *F* that measures the discrepancy between the observed means $\bar{y}$ and the model-implied means *μ*(*θ*) as well as the discrepancy between the observed covariance matrix ***S*** and the model-implied covariance matrix ***Σ**(θ)*. Several fitting functions have been proposed. The following maximum likelihood fitting function is widely used as it yields efficient parameter estimates under the assumption of multivariate normally distributed data:

(1) $$F_{ML}\left[\bar{y},S,\mu \left(\theta \right),\Sigma \left(\theta \right)\right]={\left[\bar{y}-\mu \left(\theta \right)\right]}^{\text{T}}\Sigma {\left(\theta \right)}^{-1}\left[\bar{y}-\mu \left(\theta \right)\right]+\text{tr}\left[S\Sigma {\left(\theta \right)}^{-1}\right]-\text{ ln}\left\{\text{det}\left[S\Sigma {\left(\theta \right)}^{-1}\right]\right\}-p$$

In the equation above, *p* denotes the number of observed variables in the SEM. A fitting function also provides a test of overall model fit. Evaluated at the parameter estimates $\hat{\theta }$, (*N*−1)*F* asymptotically follows a *χ*^2^ distribution with *q* degrees of freedom under the null hypothesis of a correctly specified model, where *N* refers to the sample size. A detailed account of SEM estimation can be found in the textbooks by Bollen (1989) and Kline (2016).

### Step 3: Split Evaluation

The original SEM tree algorithm suggested by Brandmaier et al. (2013b) compares the fit of a single-group model to the fit of a MGSEM, which consists of all submodels in the current leaves, to decide whether to split a node according to a covariate. For the sake of simplicity, we assume that all covariates are dichotomous and discuss non-dichotomous covariates afterward.

Let *M*_*F*_ represent the model associated with the root node (that contains the full data set) and let ${\hat{\theta }}_{F}$ denote the corresponding parameter estimates. Further, we mark the observed mean vector of the full data set as ${\bar{y}}_{F}$ and the observed covariance matrix as ***S**_*F*_*. To evaluate a candidate covariate for a specific node, we split the node into two daughter nodes according to the covariate. Then, group-specific SEM parameters *θ_*j*_*, *j* = 1, …, *J*, are estimated for all subsamples associated with the *J* current leaf nodes. Since the subsamples associated with the current leaves are non-overlapping, the submodels can be joined into a MGSEM, which we from now on refer to as *M*_*SUB*_. As *M*_*F*_ is nested within *M*_*SUB*_, we can test the following null hypothesis of parameter homogeneity with respect to the covariate under evaluation:

(2) $${\text{H}}_{0}:\;\theta_{j}=\theta_{0},\forall j=1,\ldots ,J$$

Rejecting Equation 2 implies that the model parameters vary with respect to the covariate. Brandmaier et al. (2013b) suggested using the following log-likelihood ratio between *M*_*F*_ and *M*_*SUB*_ as a test statistic for Equation 2:

(3) $$LR=\left(n-1\right)\left\{F_{ML}\left[{\bar{y}}_{F},S_{F},\mu \left({\hat{\theta }}_{F}\right),\Sigma \left({\hat{\theta }}_{F}\right)\right]-\overset{J}{\underset{j=1}{\sum }}\frac{n_{j}}{n}F_{ML}\left[{\bar{y}}_{j},S_{j},\mu \left({\hat{\theta }}_{j}\right),\Sigma \left({\hat{\theta }}_{j}\right)\right]\right\}$$

Under the null hypothesis that there is no influence of the covariate under scrutiny, *LR* asymptotically follows a *χ*^2^ distribution with (*J*−1)*q* degrees of freedom.

This testing procedure provides a powerful and efficient solution for dichotomous covariates. However, evaluating a categorical, an ordinal, or a continuous covariate that has more than two unique values requires an additional step of locating the optimal cut point. Brandmaier et al. (2013b) suggested to compute the likelihood ratio in Equation 3 for every meaningful partition of the covariate and then to select the cut point associated with the maximum likelihood ratio. For categorical covariates, the best partition is found by splitting them into a set of dichotomous variables applying a one-against-the-rest scheme for all possible combinations of categories. For ordinal and continuous covariates, the ordering inherent to these covariates allows applying a procedure known as exhaustive split search (Quinlan, 1993) to find the optimal cut point. Given a covariate with *m* unique values, this procedure tests *m*−1 potential partitions to locate the maximum of the likelihood ratios. For continuous covariates, it is also necessary to omit a certain fraction of the data associated with the smallest and largest values of the covariate in order to obtain a sufficiently large sample to estimate the SEMs in both partitions. From the above, it is clear that the computational demand of SEM trees grows with the number of covariates with many unique values as every potential cut point requires the estimation of SEMs.

Locating the optimal cut point in categorical, ordinal, and continuous covariates with the maximum of the likelihood ratios has important implications for the test statistic shown in Equation 3. By choosing the maximum of a set of statistics (one for each possible partition), the resulting distribution is no longer the same as the distribution of the individual statistics. Thus, a maximally selected likelihood-ratio test statistic does not follow a *χ*^2^ distribution under the null hypothesis of parameter homogeneity. The deviation from the *χ*^2^ distribution is directly related to the number of potential cut points. With a growing number of possible cut points, the maximum of the likelihood-ratio values will be increased purely by random fluctuations. Consequently, using the *χ*^2^ test for the evaluation of covariates will artificially inflate the probability of type I errors and favors the selection of covariates with many potential cut points over the selection of covariates with few.

Brandmaier et al. (2013b) discuss different correction procedures for this selection bias that are used in the semtree package. The default method, labeled *naïve* in semtree, uses the *χ*^2^ distribution for evaluating covariates and simply ignores the resulting selection bias. To reduce this bias, semtree offers the option to use the *naïve* method in combination with a Bonferroni correction for multiple testing within the same covariate by dividing the *p*-value obtained from the likelihood-ratio test in Equation 3 by the number of potential cut points. However, this Bonferroni adjustment can lead to overcorrection and decreases the probability of selecting covariates with many possible cut points, as demonstrated by Brandmaier et al. (2013b). We will refer to this Bonferroni adjusted *naïve* method simply as the *naïve* method from now on. Besides the Bonferroni correction, different cross-validation methods are implemented in the semtree package. Cross-validation separates the estimation of SEMs from the testing of a potential cut point (e.g., Jensen and Cohen, 2000). SEM trees can be grown with a two-stage approach (Loh and Shih, 1997; Shih, 2004; Brandmaier et al., 2013b) that splits the sample associated with a node in half. One half of the sample is used to find the optimal cut point for every covariate. The other half is used to evaluate only the best cut points via the likelihood-ratio test. This method is called *fair* in the semtree package. Since the *fair* method uses only half of the sample for split selection, its power for detecting heterogeneity can be expected to be considerably lower than the power of methods that employ the whole sample. A much simpler and more elegant way of avoiding the selection bias and correction procedures altogether is to use the correct distribution of the maximally selected likelihood-ratio test statistic (max*LR*). Andrews (1993) showed that the asymptotic distribution of max*LR* is the supremum of a certain tied-down Bessel process from which *p*-values can be obtained (see Zeileis et al., 2008; Merkle and Zeileis, 2013). We now implemented the max*LR* statistic into the semtree package to provide a more efficient and robust likelihood-ratio-based covariate selection.

### Step 4: Covariate Selection

To select a single covariate from a set of candidate covariates, the likelihood ratio for the optimal cut point is computed for every covariate, and the covariate associated with the smallest *p*-value is chosen. If the *p*-value is smaller than a pre-specified threshold, determined by the desired probability of a type I error, splitting is continued recursively. One should keep in mind that testing several covariates will artificially inflate the type I error probability. One of several solutions to this problem is the use of Bonferroni adjusted *p*-values. Given a large number of covariates, however, the Bonferroni correction will reduce the power of the SEM tree drastically and will produce sparse trees. In such cases, one may resort to unadjusted *p*-values for the selection of covariates and, if needed, can limit the size of the SEM tree with additional stopping criteria like a minimum number of individuals per node.

## Score-Guided SEM Trees

Using likelihood-ratio tests to grow SEM trees can become computationally burdensome if not infeasible as the evaluation of a covariate requires the estimation of MGSEMs for every potential cut point. Furthermore, when subgroups become small, fitting MGSEMs may become unstable. Alternatively, SEM trees can be guided by score-based tests that do not require the estimation of MGSEMs to evaluate a split at all. This makes score-based tests computationally efficient and often more stable as compared to likelihood-ratio tests. In the following, we will first introduce the general notion behind score-based tests and then introduce a family of score-based test statistics for covariates with different levels of measurement.

### Score-Based Tests

Score-based tests originated in econometrics, where they are primarily employed to detect parameter instability in time series models (e.g., Hansen, 1992; Andrews, 1993). Score-based tests can be summarized in three steps: first, the case-wise derivatives of the log-likelihood function with respect to the model parameters are computed. These case-wise derivatives, also called scores, indicate how well the model parameters represent an individual. The larger the score, the larger the misfit of a given model parameter for a given individual. Second, the scores are sorted with respect to a covariate for which we want to test parameter homogeneity. Third, the scores are aggregated into a test statistic that allows testing of the null hypothesis of homogeneous parameters (see Equation 2).

Score-based tests have been derived for general M-estimators that encompass popular estimation techniques such as least-squares methods and maximum likelihood as special cases (Zeileis and Hornik, 2007). For the sake of simplicity, we limit ourselves to maximum likelihood estimation for multivariate normally distributed data. The associated log-likelihood function for a single individual *i* is given by

(4) $$\ln L\left(\theta ;y_{i}\right)=\frac{1}{2}\left\{{\left[y_{i}-\mu \left(\theta \right)\right]}^{\text{T}}\Sigma {\left(\theta \right)}^{-1}\left[y_{i}-\mu \left(\theta \right)\right]+\ln\left[\det\left(\Sigma \left(\theta \right)\right)\right]+p\ln(2\text{π})\right\}$$

Equation 4 is the normal theory log-likelihood function for a single individual *i* and yields identical parameter estimates to *F*_*ML*_ shown in Equation 1 if summed over individuals and maximized.

The individual scores are calculated by taking the partial derivative of the log-likelihood function with respect to the parameters and evaluating the expression at the estimates:

(5) $$S\left(\hat{\theta };y_{i}\right)={\left[\begin{array}{ccc} \frac{\partial \ln L\left(\theta ;y_{i}\right)}{\partial \theta_{1}}{│}_{\theta =\hat{\theta }} & \cdots & \frac{\partial \ln L\left(\theta ;y_{i}\right)}{\partial \theta_{q}}{│}_{\theta =\hat{\theta }} \end{array}\right]}^{\text{T}}$$

The scores assess the extent to which an individual’s log-likelihood is maximized by one of the *q* parameters. Values close to zero indicate a good fit between model and individual, whereas large scores point toward a strong misfit. Note that by definition, the scores evaluated at the maximum likelihood estimates $\hat{\theta }$ sum up to zero; that is, $\overset{n}{\underset{i=1}{\sum }}S\left(\hat{\theta };y_{i}\right)=0$.

For the construction of a test statistic, the scores are cumulated according to the order induced by a covariate under scrutiny. For instance, if parameter homogeneity is assessed with respect to age, the first row consists of scores from the youngest individual. For the second row, scores of the youngest and second youngest individuals are summed up, and so forth. More formally, the cumulative score process is defined as

**FIGURE 2:**
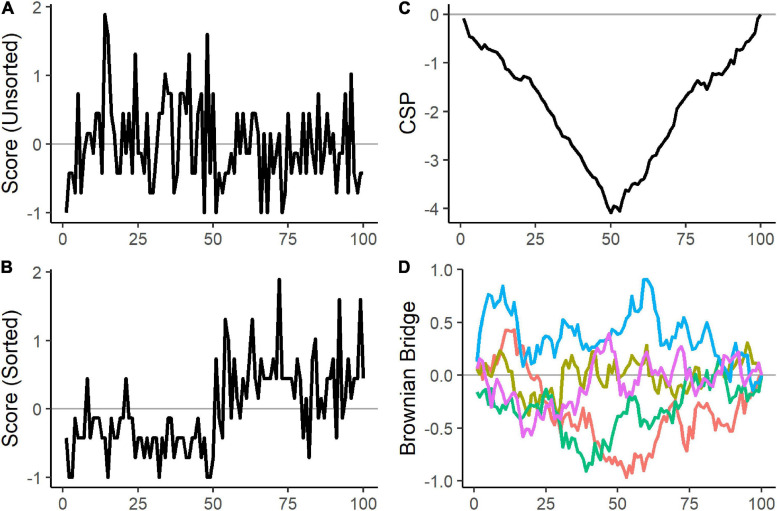
Artificial example to visualize the effect of sorting and cumulating. 100 observations were sampled from two Poisson distributions with different rate parameters. 50 observations were generated with a rate parameter of 2 and 50 observations with a rate parameter of 5. Panel **(A)** shows the scores of the 100 observations in random order. Panel **(B)** displays the 50 scores of observations generated with a rate parameter of 2 first, followed by the 50 scores sampled with a rate parameter of 5. After sorting the scores according to the two groups, a clear pattern emerges as the first 50 scores are mostly negative, and the remaining scores are mostly positive. Panel **(C)** shows the cumulative score process. The negative and positive scores are cumulated, and the change point is noticeable from the negative peak in the cumulative score process. Panel **(D)** depicts five randomly generated Brownian bridges. Under the null hypothesis of a constant rate parameter, the cumulative score process would have behaved similarly to the 5 Brownian bridges in Panel **(D)**.

(6) CSP(θ^;s)=1n I(θ^)−12∑h=1sS(θ^;yh)

where the index *s* denotes the number of sorted individuals entering the equation, and the index *h* selects the sorted individuals until *h* = *s*. Furthermore, I(θ^)−12 is the estimated half-squared inverse of the Fisher information matrix. Pre-multiplying with I(θ^)−12 decorrelates the scores so that the *q* cumulative score processes are unrelated to each other. In the following, we place the values of the cumulative score process row-wise into an *N* × *q* matrix that we denote with ***CSP*** and refer to the cumulative sum from the first *s-*th individuals of the *k*-th parameter as *CSP*_*s,k*_. The plots in Panel (A–C) in Figure 2 illustrate how sorting and cumulating scores make parameter heterogeneity visible.

Hjort and Koning (2002) show that under mild conditions and constant parameters, each column of the cumulative score process matrix ***CSP*** converges in distribution to a univariate Brownian bridge. A Brownian bridge is a stochastic process that is pinned to zero at the start and end and exhibits the most variability in the middle. Thus, the null hypothesis of parameter homogeneity in Equation 2 can be tested by comparing the observed cumulative score process to the analogous statistic of a Brownian bridge. Panel (C) and (D) in Figure 2 illustrate the difference between the cumulative score process of a heterogeneous parameter and the Brownian bridge.

Test statistics can be obtained by aggregating the cumulative score process matrix into a single scalar. Critical values and *p*-values for these test statistics can be found by applying the same aggregation to the asymptotic Brownian bridge (Zeileis and Hornik, 2007). Different ways of aggregating the cumulative scores will produce test statistics that will be sensitive to different patterns of parameter heterogeneity. The choice of a test statistic also depends on the level of measurement of the covariate.

Merkle and Zeileis (2013) proposed three different test statistics for continuous covariates:

(7) $$D\,M=\max_{s=1,\ldots ,N}\left[\underset{k=1,\ldots \,q}{\text{max}}\left(\left|C\,S\,P_{s,k}\right|\right)\right]$$

(8) $$C\,v\,M=\frac{1}{N}\,\sum_{s=1}^{N}\sum_{k=1}^{q}C\,S\,P_{s,k}^{2}$$

(9) $$\text{max}\,L\,M=\max_{s=\underline{s},\ldots ,\bar{s}}\left\{{\left[\frac{s}{N}\,\left(1-\frac{s}{N}\right)\right]}^{-1}\,\sum_{k=1}^{q}C\,S\,P_{s,k}^{2}\right\}$$

Equations 7–9 show the double maximum (*DM*), Cramér-von-Mises (*CvM*), and maximum Lagrange multiplier (max*LM*) test statistics. *DM* is the simplest test statistic and rejects the null hypothesis if, at any point, the maximum of any of the *q* processes strays too far away from zero. However, Merkle and Zeileis (2013) note that considering only the maximum of the *q* processes wastes power because the *DM* statistic ignores heterogeneity in other parameters. Furthermore, even for the same parameter, smaller peaks before and after the maximum are not considered, which may lead to a loss of power if the parameter changes its values across more than two groups. Using sums instead of maxima solves these problems. The *CvM* statistic sums the squared values over all parameters and individuals and is therefore well suited for detecting multiple group differences in several parameters. If one suspects that a single change point will manifest in several parameters, the max*LM* statistic that considers the maximum values of all parameters at a single point is more appropriate. Unlike the other test statistics for continuous covariates, the max*LM* statistic contains a scaling term $\frac{s}{N}\,\left(1-\frac{s}{N}\right)$, which increases sensitivity for peaks before and after the middle of the processes. A disadvantage of this scaling is that individuals with very small and very large values of the covariate need to be omitted to stabilize the test statistic. Therefore, one has to specify an interval $\left[\underline{s},\ldots ,\bar{s}\right]$ with a lower and upper threshold of the covariate. Parameter shifts outside of these boundaries are not considered. The max*LM* statistic is asymptotically equivalent to the max*LR* statistic from the previous section (Andrews, 1993).

For ordinal and categorical covariates, Merkle et al. (2014) suggested test statistics that focus on bins of individuals at each level of the covariates:

(10) $$W\,D\,M=\max_{l=1,\ldots ,m-1}\left\{{\left[\frac{n_{l}}{N}\,\left(1-\frac{n_{l}}{N}\right)\right]}^{-1/2}\,\max_{k=1,\ldots \,q}\left|C\,B\,S\,P_{l,k}\right|\right\}$$

(11) $$\text{max}\,L\,M_{O}=\max_{l=1,\ldots ,m-1}\left\{{\left[\frac{n_{l}}{N}\,\left(1-\frac{n_{l}}{N}\right)\right]}^{-1}\,\sum_{k=1}^{q}C\,B\,S\,P_{l,k}^{2}\right\}$$

Equations 10 and 11 present the weighted double maximum (*WDM*) and the maximum Lagrange multiplier statistics for ordinal covariates (max*LM*_*O*_). For both test statistics, we first group the individuals into *m*−1 bins associated with the first *m*−1 levels of the covariate. Then, we sum the scores in each bin and cumulate the sums, yielding a (*m*−1) × *q* matrix ***CBSP*** of cumulative bins of scores. In the equations above, we denote the cumulative bin of scores associated with the *l*-th level of the covariate and the *k*-th parameter with *CBSP*_*l,k*_. Both statistics are scaled by $\frac{n_{l}}{N}\,\left(1-\frac{n_{l}}{N}\right)$, where *n*_*l*_ represents the cumulative number of individuals per bin. The main difference is that the max*LM*_*O*_ statistic considers heterogeneity in all parameters, whereas the *WDM* only considers the most heterogeneous parameter.

Categorical covariates do not possess a natural ordering that can be used to construct a test statistic. Alternatively, a test statistic can be obtained by summing the squared differences in the sum of scores across bins of individuals associated with a different level of the covariate (Hjort and Koning, 2002). In the following Lagrange multiplier (*LM*) statistic

(12) $$L\,M=\sum_{l=1}^{m}\sum_{k=1}^{q}{\left(B\,S\,P_{l,k}-B\,S\,P_{l-1,k}\right)}^{2},$$

*BSP*_*l,k*_ denotes the sum of the scores of the *k*-th parameter from individuals associated with the *l*-th level of the covariate. *B*_0,_*_*k*_*, *k* = 1, …, *q*, is not associated with any of the 1, …, *m* levels of the covariate and is set to zero.

To apply the test statistics outlined above in practice, critical values and *p*-values are needed in order to compare split points across covariates. Analytic solutions are available for the *DM*, max*LR*, *WDM*, and *LM* statistic. For the remaining test statistics, critical values and *p*-values can be obtained through repeated simulation of Brownian bridges. Different strategies for obtaining critical values and *p*-values for the *DM*, max*LR*, and *CvM* statistics are discussed by Merkle and Zeileis (2013) and for the *WDM*, max*LM_*O*_*, and *LM* statistics by Merkle et al. (2014).

### SEM Trees Guided by Score-Based Tests

Score-guided SEM trees can be obtained by replacing the evaluation of covariates in Step 3 of the general SEM tree algorithm with score-based tests instead of the likelihood-ratio test. Because score-based tests operate like an omnibus test for all possible cut points in a covariate, a single best cut point needs to be located after the selection of a covariate. Cut points can be obtained by identifying which of the unique values of the covariate maximizes the respective score-based test statistic. Omitting the outer sums or maxima in the Equations 7–11 pairs every unique value of the covariate with a specific value of the partially summed test statistic. Then, a cut point can be determined by splitting the sample after the observation associated with the maximum of these partially summed test statistics. Due to its scaling term, the respective max*LM* statistic for ordinal and continuous covariates appears to be particularly well suited for identifying the optimal cut points. We implemented this fully score-based cut point localization procedure in the semtree package. Alternatively, the optimal cut point can be determined by maximizing the partitioned log-likelihood (that is, the sum of the log-likelihood for all observations to the left and the sum for all observations to the right of the cut point) over all conceivable values of the covariate. Since this approach requires the estimation of a sequence of SEMs, it will be slower than a purely score-based cut point identification. However, it will still be faster than a SEM tree purely guided by likelihood ratios because only the localization of a cut point but not the selection of the covariate requires the estimation of additional SEMs. This hybrid strategy is currently applied by the generic MOB algorithm from the partykit package, which uses the max*LM* statistic for selecting covariates and the max*LR* statistic for locating cut points.

## Simulation Study

We conducted four Monte Carlo simulations to evaluate SEM trees in different settings. The first two simulations compare the original SEM tree split selection methods with the newly proposed SEM trees guided by the max*LR* statistic and score-based tests. The first simulation aims at illustrating the performance of the different SEM trees under the null hypothesis of parameter homogeneity. The second simulation investigates power, the precision of cut point estimation, and group recovery for a heterogeneous population consisting of two groups. The third and fourth simulations demonstrate the use and common pitfalls of SEM trees with focus parameters and equality constraints.

All simulations were carried out with the statistical programming language R. SEM trees were fitted with the semtree package. semtree interfaces the OpenMx package (Neale et al., 2016) for the estimation of SEMs. To grow score-guided SEM trees, we linked semtree to the strucchange package (Zeileis et al., 2002). strucchange offers a unified framework for implementing score-based tests for a wide range of models. All features used in this simulation are available in the semtree package. Our simulations were performed using R 4.0.2, OpenMx 2.18.1, strucchange 1.5–2, and a developmental snapshot of the semtree package^1^. The simulation scripts and results are provided as Online Supplemental Material^2^.

In all simulations, we aimed at ensuring an optimal type I error rate; that is, we tried limiting the proportions of false-positive splits to the significance level of 5%. To achieve this, we adjusted the *p*-values of the likelihood-ratio and score-based tests with the Bonferroni procedure to correct for the multiple testing of several covariates. Besides the Bonferroni correction, we used the default settings of the semtree package throughout our simulation studies. The score-based tests were performed by applying the default settings of the strucchange package. The data used to fit the SEMs were drawn from a multivariate normal distribution. All experimental conditions were replicated 10,000 times.

### Simulation I: Type I Error Rate and Runtime

Simulation I assessed the type I error rate under the null hypothesis of constant parameters and the runtime for a different number of noise variables and sample sizes. The simulated data was homogeneous without any group differences.

**FIGURE 3:**
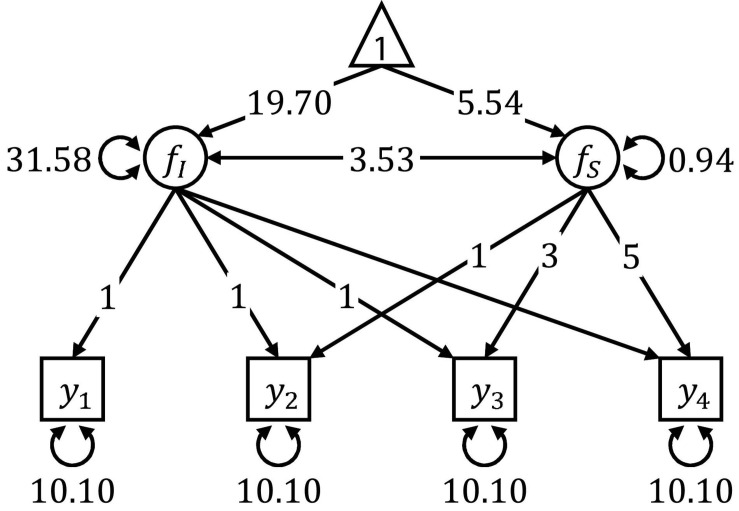
Path diagram of the linear latent growth curve model used for data generation in Simulation I and II. The parameter values were obtained by fitting the model on scores from the longitudinal Wechsler Intelligence Scale for Children data set.

Figure 3 shows the linear latent growth curve model used in Simulation I and II. Model specification and parameter values were taken from McArdle and Epstein (1987), who modeled the scores of 204 young children from the Wechsler Intelligence Scale for Children over four repeated occasions of measurement at 6, 7, 9, and 11 years of age (see Brandmaier et al., 2013b for a SEM tree analysis of these data). In both simulation studies, we generated multivariate normal data, using the mean vector and covariance matrix implied by the model presented in Figure 3.

After generating the data, the linear latent growth curve model presented in Figure 3 was estimated, serving as a template model for the SEM trees. The model was defined by six free parameters: the mean and the variance of the random intercept *f*_*I*_, the mean and the variance of the random slope *f*_*S*_, the covariance between the random intercept and the random slope, and the residual error variance that was constrained to be equal for all four measurements of the observed variable *y*.

The following experimental factors were varied:

• *Level of measurement of the noise variables*: We provided the SEM trees with randomly generated noise variables. The noise variables were either continuous (standard normal), ordinal with 6 levels (with an equal number of observations per level), or dichotomous (with an equal number of observations in both classes). For a given condition, all noise variables had the same level of measurement.
• *Number of noise variables*: Either 1, 3, or 5 noise variables were generated.
• *Sample size (N)*: The simulated samples contained either 504 or 1,008 observations. The odd numbers resulted from the necessity to be divisible by 6 to allow for an equal number of observations per level of the ordinal noise variables.

First, we will inspect the type I error rates of the different SEM tree approaches and compare their computation time afterward.

#### Percentage of Type I Errors

Every tree consisting of more than one node was counted as a type I error. Ideally, the proportion of type I errors should approach 5%. Table 1 shows the empirical type I error rates of the different SEM tree approaches. The results are sorted with respect to the level of measurement of the noise variables. To get a better understanding of the simulated error rates, we printed results for methods that fell inside a 95% confidence interval around the optimal rate of 5% for 10,000 replications (CI: [4.573; 5.427]) in bold. For ordinal and dichotomous noise variables, all SEM tree implementations yielded error rates mostly close to the desired 5%. For continuous covariates, however, only *fair*, max*LR*, *CvM*, and max*LM* trees had satisfactory type I error rates. *DM* trees exhibited slightly too few type I errors. As predicted by Brandmaier et al. (2013b), *naïve* trees that were provided with continuous noise variables over-adjusted and produced error rates that were too small by a factor of 10. Increasing the sample sizes amplified this overcorrection. For the remaining methods, varying the number of noise variables and the sample size did not systematically influence the error rates.

**TABLE 1:** Empirical type I error rates.

		Continuous						Ordinal					Dichotomous		
Nr. noise	*N*	*Naïve*	*Fair*	max*LR*	*DM*	*CvM*	max*LM*	*Naïve*	*Fair*	max*LR*	*WDM*	max*LM*_*O*_	*Naïve*	*Fair*	*LM*
1	504	0.56	**5.15**	**5.27**	3.85	**5.01**	**5.05**	4.50	5.71	**5.31**	**5.08**	**5.16**	**5.15**	**5.21**	**4.89**
3	504	0.60	**5.01**	5.43	4.17	**5.05**	5.57	**4.99**	5.78	**5.41**	**5.38**	5.59	**5.18**	5.45	**4.76**
5	504	0.51	**5.26**	**5.25**	4.18	**5.11**	5.62	**5.10**	5.73	5.66	5.59	5.69	**5.07**	**5.39**	4.55
1	1,008	0.25	**4.71**	**5.39**	3.93	**4.86**	**5.17**	3.93	**5.27**	**4.71**	**4.95**	**4.76**	**5.17**	**5.31**	**4.99**
3	1,008	0.35	**4.95**	**5.04**	3.91	4.57	**5.01**	**4.80**	5.67	**5.23**	5.60	**5.24**	**5.13**	**4.97**	**4.92**
5	1.008	0.35	**5.23**	**5.37**	**4.61**	**5.13**	5.48	**4.95**	5.60	**5.27**	**5.25**	5.68	**4.81**	**5.04**	**4.61**

*Nr. noise = number of noise variables, *N* = sample size. Error rates within the 95% confidence interval around the optimal rate of 5% are printed in bold.*

#### Runtime

We recorded the computation time for the different SEM trees in seconds. As a matter of course, the runtime varies widely depending on the computing platform. However, comparing the runtime of the different methods allows for relative comparisons and provides estimates for current standard computing platforms. Necessarily, the absolute estimates will become outdated soon. The simulation was conducted with an Intel^®^ Xeon^®^ CPU E5-2670 processor using a single core.

Table 2 presents the median of the computation time in seconds. The median runtime for ordinal and dichotomous noise variables was small. The score-guided trees (*WDM*, max*LM*_*O*_, and *LM*) showed a minor speed advantage over the likelihood-ratio-guided trees (*naïve*, *fair*, and max*LR*). For continuous noise variables, however, for which many possible cut points needed to be evaluated, the runtime of likelihood-ratio-guided SEM trees was excessively larger than the computation time of score-guided SEM trees. For instance, given the larger sample size and five noise variables, likelihood-ratio-guided SEM trees needed several minutes to compute, whereas score-guided SEM trees (*DM*, *CvM*, and max*LM*) were performed in fractions of a second. The runtime of the *fair* trees was roughly half as long as the runtime of *naïve* and max*LR* trees, most likely because the *fair* method tests only half of the possible cut points for continuous variables. As expected, a larger sample size and more noise variables led to an increase in computation time of the likelihood-ratio-guided SEM trees. In contrast, the runtime of score-guided SEM trees remained virtually the same. This implies that even for larger samples consisting of larger numbers of individuals and many covariates, score-guided SEM trees can be computed in short time.

**TABLE 2:** Median runtime in seconds.

		Continuous						Ordinal					Dichotomous		
Nr. noise	*N*	*Naïve*	*Fair*	max*LR*	*DM*	*CvM*	max*LM*	*Naïve*	*Fair*	max*LR*	*WDM*	max*LM*_*O*_	*Naïve*	*Fair*	*LM*
1	504	35.0	15.8	34.7	0.2	0.2	0.2	0.6	0.7	1.4	0.2	0.2	0.4	0.4	0.2
3	504	105.6	48.1	105.7	0.2	0.2	0.2	1.3	1.5	1.9	0.2	0.2	0.4	0.7	0.2
5	504	179.3	81.0	179.3	0.2	0.2	0.2	2.1	2.5	2.6	0.2	0.2	0.7	1.0	0.2
1	1,008	72.7	34.5	72.7	0.2	0.2	0.2	0.5	0.6	1.3	0.2	0.2	0.3	0.4	0.2
3	1,008	222.7	105.0	222.7	0.2	0.2	0.2	1.3	1.5	1.9	0.2	0.2	0.5	0.7	0.2
5	1.008	374.7	175.4	366.7	0.2	0.2	0.2	2.1	2.5	2.7	0.3	0.3	0.8	1.1	0.2

*Nr. noise = number of noise variables, *N* = sample size.*

### Simulation II: Power, Cut Point Estimation, and Group Recovery

Simulation II evaluated the performance of likelihood-ratio and score-guided SEM trees in heterogeneous samples consisting of two subgroups.

We varied the following experimental factors:

• *Level of measurement of the covariate:* The SEM tree was provided with a single covariate that was either a continuous variable (standard normal), an ordinal variable with 6 levels, or a dichotomous variable.
• *Group differences:* We tested two types of group differences. Either the fixed slope of the linear latent growth curve model shown in Figure 3 or all random effects varied between groups. Table 3 presents the values used for the heterogeneous parameters. Note that in the fixed slope condition, only a single parameter varied between groups, whereas in the random effects condition, three parameters varied. The values of the remaining homogeneous parameters are shown in Figure 3.

**TABLE 3:** Parameter differences used in Simulation II.

	Fixed effects			Random effects	
Parameter	Group 1	Group 2	Parameter	Group 1	Group 2
E(*f*_*I*_)	5.389	5.695	Var(*f*_*I*_)	25.137	38.023
			Var(*f*_*S*_)	2.808	4.247
			Cov(*f*_*I*_,*f*_*S*_)	0.745	1.127

• *Noise variable*: In the noise condition, the SEM tree algorithm was provided with a noise variable in addition to the informative covariate. In the no-noise condition, only the informative covariate was given to the tree. The noise variable was independent of the group differences and randomly selected to be a continuous variable (standard normal), an ordinal variable with 6 levels, or a dichotomous variable.
• *Cut point location:* We tested three different positions of the optimal cut point in the informative covariate. The cut points were either central, partitioning the sample into two groups of equal size, moderately non-central, resulting in a larger subgroup consisting of 66.67% of the observations and a smaller subgroup with 33.33% of the observations, or strongly non-central with 83.33% of the observations in the larger subgroup and 16.67% of the observations in the smaller subgroup. We counterbalanced the non-central cut points so that moderately non-central cut points occurred either after the 13- or after the 23-quantile of the covariate and strongly non-central cut points either after the 16- or the 53-quantile.
• *Sample size (N)*: The sample consisted either of 504 or 1,008 observations.

We evaluated each method in terms of statistical power to detect heterogeneity, the precision of the estimated cut points, group recovery, and runtime. For each condition, the results of the best-performing method are printed in bold in the following tables. Due to space constraints, we report only the most important simulation results. The complete simulation results are provided as Online Supplemental Material^2^.

#### Power

We define statistical power as the percentage of SEM trees that correctly selected the covariate as a split at any cut point and any level of the tree.

Table 4 shows the estimated power of the different SEM trees. We will first compare the overall performance of the original *naïve* and *fair* trees with the newly implemented max*LR* and score-guided trees. With respect to power, we found that *naïve* trees performed roughly as well as the newly implemented methods for ordinal and dichotomous covariates but poorly for continuous covariates. The other classical method, *fair* trees, showed overall the lowest power of all methods under investigation. As expected, the likelihood-ratio-guided max*LR* trees yielded similar results as the score-guided max*LM* trees but were consistently slightly more powerful. Among the experimental conditions, the type of group differences and the cut point location impacted the rank order of the methods the most. *DM* and *WDM* trees were the most powerful methods for detecting heterogeneity in the fixed slope parameter. In contrast, max*LR*, *CvM*, max*LM*, and max*LM*_*O*_ trees proved to be the more powerful methods for detecting heterogeneity in the random effects. We expected this behavior because the *DM* and *WDM* test statistics focus on heterogeneity in a single parameter, whereas all other methods monitor group differences in multiple parameters. Overall, the likelihood-ratio-based test statistic max*LR* and the score-based test statistics with a scaling term (that is, max*LM*, *WDM*, and max*LM*_*O*_) were more sensitive for non-central cut points but less sensitive for central cut points than the *DM* and *CvM* statistics for continuous covariates that do not use any scaling. As an optimal baseline, we compared the power of the SEM trees with MGSEMs, denoted as MG in Table 4. Like the SEM trees, the MGSEMs were specified by letting all parameters vary between groups. In contrast to SEM trees, MGSEMs were unaffected by noise variables and were informed about the true cut point. Therefore, the MGSEMs present the upper limit achievable in terms of statistical power. Not surprisingly, MGSEMs were more powerful than all SEM tree methods, given continuous and ordinal covariates, but equally powerful in conditions with dichotomous covariates and without noise variables, where cut points did not need to be learned from the data.

**TABLE 4:** Power to detect group differences.

		Continuous							Ordinal						Dichotomous			
*N*	CL	*Naïve*	*Fair*	max*LR*	*DM*	*CvM*	max*LM*	*MG*	*Naïve*	*Fair*	max*LR*	*WDM*	max*LM*_*O*_	*MG*	*Naïve*	*Fair*	*LM*	*MG*
***Group difference in the fixed slope***																		
504	1/2	10.0	14.5	36.8	**46.7**	41.7	35.4	52.8	35.8	17.4	38.4	**44.2**	37.0	52.4	**52.8**	27.0	51.8	52.8
	1/3	7.7	13.1	31.6	**35.4**	32.4	30.5	47.1	30.4	15.7	32.6	**37.7**	31.6	46.1	**45.9**	24.0	45.2	45.9
	1/6	3.2	8.1	**16.8**	9.8	13.0	16.4	29.7	17.8	10.4	19.4	**21.2**	19.0	29.8	**30.1**	17.0	29.3	30.1
1,008	1/2	30.6	29.8	72.9	**84.9**	76.3	72.1	87.2	73.8	37.6	75.7	**83.3**	75.0	86.4	**85.5**	51.4	85.2	85.5
	1/3	24.3	26.1	66.1	**74.9**	64.8	65.6	82.0	65.7	32.3	68.0	**77.2**	67.4	81.3	**81.5**	47.6	81.2	81.5
	1/6	8.7	15.6	**38.5**	25.9	26.2	**38.5**	58.2	39.5	19.9	42.0	**48.7**	41.4	57.9	58.6	30.4	**58.7**	58.6
***Group differences in the random effects***																		
504	1/2	18.2	19.4	51.8	48.3	**56.5**	49.9	69.4	52.1	24.5	**54.2**	46.4	53.2	68.5	**68.8**	36.0	67.4	68.8
	1/3	13.7	17.1	44.1	35.6	**44.6**	42.8	62.8	45.0	21.0	**47.5**	39.9	45.9	63.1	**62.9**	32.8	61.0	62.9
	1/6	5.3	10.4	22.9	12.0	18.1	**23.8**	40.6	24.5	13.4	26.4	22.8	**26.8**	40.9	**41.1**	21.0	38.0	41.1
1,008	1/2	53.3	45.1	88.0	85.7	**89.6**	87.6	95.9	89.4	55.0	**90.4**	84.2	90.1	95.7	**95.7**	69.1	95.5	95.7
	1/3	43.9	37.5	**82.7**	74.4	80.0	80.9	93.3	84.2	47.7	**85.6**	77.5	84.3	93.1	**93.4**	63.3	93.0	93.4
	1/6	17.3	21.0	**52.8**	26.6	36.4	49.9	73.4	55.3	28.3	**57.4**	47.3	53.6	73.4	**73.3**	39.8	69.8	73.3

**N* = sample size, CL = cut point location, 1/2 = central cut point location, 1/3 = moderately non-central, 1/6 = strongly non-central, *MG* = MGSEM. Best-performing methods are printed in bold.*

The presence of a noise variable (not shown in Table 4) approximately halved the power of all tree methods but affected *naïve* trees most severely. The pronounced effect of noise variables on *naïve* trees was mainly driven by continuous noise variables, which led to severely over-adjusted *p*-values. Providing *naïve* trees with ordinal or dichotomous noise variables led to a decrease of power that was comparable to the decrease in other methods. Increasing the sample size had an approximately uniform effect and raised the power of all methods substantively.

#### Precision of Estimated Cut Points

The estimation of the optimal cut point in the covariate is crucial for recovering the true grouping of individuals. The approaches for locating cut points differed between likelihood-ratio and score-guided SEM trees. Likelihood-ratio-guided trees found cut points by maximizing a partitioned log-likelihood, and score-guided SEM trees determined cut points by searching through a disaggregated max*LM* statistic. We limit ourselves to discuss cut points estimated by max*LR* and max*LM* trees in the following. We used only trees that selected the covariate for the initial split of the data, ignoring possible further splits. Since noise variables had no visible effect, we will discuss only simulation trials without additional noise variables. Also, we did not evaluate dichotomous covariates because there is only a single trivial cut point.

Table 5 presents bias, standard deviation, and root mean squared error (RMSE) of the estimated cut points. Both approaches produced similar cut points that were nearly unbiased. Overall, cut points estimated by max*LR* were slightly more precise in terms of RMSE. Interestingly, group differences in the random effects led to slightly biased cut point estimates provided by max*LM* trees, which was not observed for max*LR* trees. Estimates for non-central cut points showed more variability than for central cut points in both methods. A larger sample size of 1,008 observations increased both methods’ precision and reduced the bias of cut points estimated by max*LM*.

**TABLE 5:** Estimated cut points.

		Continuous						Ordinal					
		max*LR*			max*LM*			max*LR*			max*LM*_*O*_		
*N*	CL	B	SD	RMSE	B	SD	RMSE	B	SD	RMSE	B	SD	RMSE
***Group difference in the fixed slope***													
504	1/2	0.013	0.388	0.389	0.017	0.408	0.408	−0.003	0.798	0.797	0.011	0.750	0.750
	1/3	0.014	0.430	0.430	0.014	0.440	0.440	0.022	0.955	0.956	0.013	0.878	0.878
	1/6	0.011	0.694	0.694	−0.022	0.686	0.686	0.010	1.347	1.347	0.015	1.312	1.312
1,008	1/2	−0.002	0.273	0.273	0.000	0.282	0.282	−0.004	0.546	0.545	−0.010	0.459	0.459
	1/3	−0.006	0.310	0.310	−0.007	0.311	0.311	−0.004	0.603	0.603	−0.006	0.494	0.494
	1/6	0.004	0.491	0.491	−0.001	0.499	0.499	0.004	0.933	0.933	0.007	0.836	0.836
***Group differences in the random effects***													
504	1/2	0.014	0.345	0.345	0.183	0.348	0.393	0.003	0.699	0.699	0.255	0.790	0.831
	1/3	0.011	0.381	0.381	0.177	0.401	0.439	0.018	0.769	0.769	0.246	0.882	0.916
	1/6	0.013	0.611	0.611	0.098	0.602	0.610	0.003	1.187	1.187	0.138	1.260	1.267
1,008	1/2	0.015	0.224	0.225	0.085	0.240	0.255	0.001	0.415	0.415	0.118	0.542	0.555
	1/3	0.011	0.245	0.245	0.092	0.268	0.283	0.010	0.457	0.457	0.117	0.600	0.611
	1/6	0.004	0.387	0.387	0.076	0.421	0.428	−0.001	0.728	0.728	0.102	0.943	0.949

**N* = sample size, CL = cut point location, 1/2 = central cut point location, 1/3 = moderately non-central, 1/6 = strongly non-central, B = bias, SD = standard deviation, RMSE = root mean squared error.*

#### Group Recovery

We used the adjusted Rand index (ARI; Hubert and Arabie, 1985; Milligan and Cooper, 1986) to measure how well the true groups are recovered by each SEM tree method. The ARI is widely used to measure the similarity between two partitions and is adjusted for agreement by chance. A large ARI value up to the maximum of 1 indicates a high agreement between the partitioning estimated by a tree and the true partitioning, while smaller values imply a lower degree of similarity. Particularly, an ARI of 0 is obtained if a tree fails to detect any group differences and does not split the sample.

The ARI of the different tree methods is shown in Table 6. In our simulation setup, the ARI of a SEM tree method seemed to be mainly determined by its power to detect heterogeneity as a similar rank order as for the statistical power emerged. Given a continuous covariate, score-guided *DM* and *CvM* trees showed the largest ARI for central cut points, max*LM* and max*LR* trees showed the largest ARI for non-central cut points, while the original likelihood-ratio-guided *naïve* and *fair* trees performed poorly. As with power, *DM* trees had a higher ARI for a difference in the slope, and the ARI of the other score-guided and max*LR* trees was higher for differences in the random effects. *Naïve* trees performed better when provided with an ordinal or dichotomous covariate. For ordinal covariates, *WDM* trees exhibited the largest ARI if the fixed slope differed between groups, whereas the ARI of max*LR* and max*LM*_*O*_ trees was higher for differences in the random effects. For dichotomous covariates, *naïve* trees showed a slightly higher ARI than score-guided *LM* trees. However, if provided with an additional noise variable, *naïve* trees showed a more pronounced decrease in the ARI than *LM* trees (not shown in Table 6). This effect was mainly driven by continuous noise variables, which led to overcorrected *p*-values of *naïve* trees. Non-central cut points generally reduced the ARI of all trees, affecting *DM* and *CvM* trees without a scaling term the most. The ARI of all tree methods improved substantially for larger samples with 1,008 simulated individuals without drastically changing the rank order.

**TABLE 6:** Adjusted Rand index.

		Continuous						Ordinal					Dichotomous		
*N*	CL	*Naïve*	*Fair*	max*LR*	*DM*	*CvM*	max*LM*	*Naïve*	*Fair*	max*LR*	*WDM*	max*LM*_*O*_	*Naïve*	*Fair*	*LM*
***Group difference in the fixed slope***															
504	1/2	0.069	0.085	0.243	**0.377**	0.323	0.234	0.277	0.120	0.296	**0.361**	0.294	**0.527**	0.270	0.518
	1/3	0.055	0.075	0.208	**0.261**	0.215	0.204	0.230	0.108	0.246	**0.306**	0.248	**0.459**	0.240	0.452
	1/6	0.022	0.044	0.101	0.036	0.036	**0.105**	0.131	0.065	0.141	**0.159**	0.142	**0.301**	0.170	0.293
1,008	1/2	0.248	0.209	0.557	**0.726**	0.636	0.556	0.647	0.313	0.662	**0.751**	0.675	**0.855**	0.514	0.852
	1/3	0.196	0.179	0.500	**0.604**	0.490	0.504	0.575	0.267	0.593	**0.697**	0.607	**0.815**	0.476	0.812
	1/6	0.067	0.098	0.275	0.137	0.097	**0.290**	0.335	0.159	0.353	**0.430**	0.358	0.586	0.304	**0.587**
***Group differences in the random effects***															
504	1/2	0.136	0.125	0.361	0.378	**0.441**	0.341	0.428	0.187	**0.444**	0.361	0.413	**0.688**	0.360	0.674
	1/3	0.102	0.109	0.309	0.252	**0.312**	0.293	0.371	0.160	**0.388**	0.308	0.356	**0.629**	0.328	0.610
	1/6	0.038	0.063	0.150	0.049	0.066	**0.170**	0.192	0.091	0.205	0.175	**0.206**	**0.411**	0.210	0.380
1,008	1/2	0.449	0.339	0.711	0.714	**0.763**	0.701	0.818	0.479	**0.827**	0.747	0.800	**0.957**	0.691	0.955
	1/3	0.370	0.281	**0.664**	0.585	0.632	0.645	0.769	0.415	**0.780**	0.686	0.744	**0.934**	0.633	0.929
	1/6	0.142	0.149	**0.406**	0.141	0.171	0.398	0.494	0.243	**0.509**	0.404	0.457	**0.733**	0.398	0.698

**N* = sample size, CL = moderately non-central, 1/2 = central cut point location, 1/3 = moderately non-central, 1/6 = strongly non-central, *MG* = MGSEM. Best-performing methods are printed in bold.*

#### Runtime

The computation time of the SEM trees in Simulation II was in line with the observed runtime in Simulation I. Overall, the median computation time of score-guided SEM trees was 0.50 s with little variability across the simulation conditions. In contrast, the runtime of likelihood-ratio-guided trees varied considerably according to the level of measurement of the covariate and noise variable. The overall median computation time for simulation conditions with ordinal or dichotomous covariate and noise variable was 0.88 s for *naïve* trees, 0.89 s for *fair* trees, and 1.40 s for max*LR* trees. However, if either the covariate or the noise variable were continuous, the overall median runtime increased drastically. For instance, for conditions with a continuous covariate, a continuous noise variable, and a sample size of 504, the computation time of *naïve*, *fair*, and max*LR* trees increased to 70.18, 32.85, and 71.05 s, respectively. For samples with 1,008 individuals, the runtime increased to 149.91, 72.23, and 280.58 s, respectively.

### Simulation III: Focus Parameters

The goal of Simulation III was to demonstrate how specific hypotheses about parameter heterogeneity, such as certain types of measurement invariance, can be tested with the use of SEM trees with focus parameters. By default, SEM trees split with respect to differences in any model parameter. At times, researchers may be interested in finding group differences only for a subset of parameters that are referred to as focus parameters in the semtree package. When focus parameters are given, SEM trees will only assess heterogeneity in these parameters and ignore group differences in the remaining parameters to evaluate a split.

**FIGURE 4:**
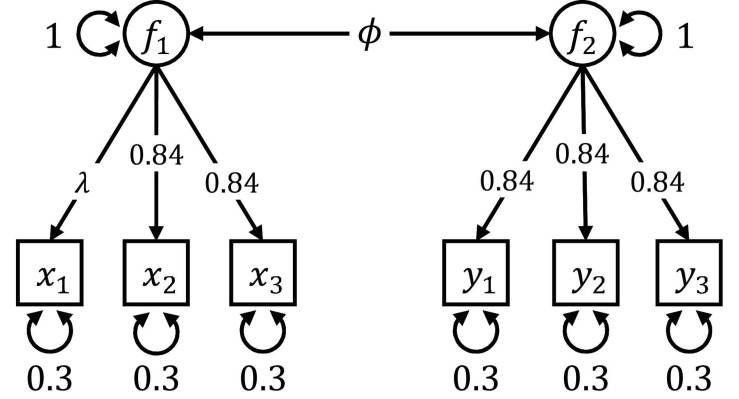
Path diagram of the CFA model used in Simulation III and Simulation IV. The model consists of two correlated latent factors, each measured by three indicators. The latent covariance *ϕ* differed between the two groups in Simulation III. In Simulation IV, there were also differences in the factor loading *λ*.

Figure 4 shows the population model used to generate data in Simulation III and IV. Depicted is a confirmatory factor model with two correlated factors, each measured by three indicators. A two group-population was simulated, where both factors were uncorrelated in the first group (*ϕ*_*g*1_ = 0) and covaried with *ϕ*_*g*2_ = 0.471 in the second group. The factor loading *λ* did not vary in Simulation III and was set to 0.837 in both groups, corresponding to a latent factor accounting for 70% of the variance in the observed variables. In each simulation replication, we generated 250 individuals per group, resulting in a total sample size of 500. The model shown in Figure 4 was then estimated with a common identification constraint; specifically, by fixing the variances of the two factors *f*_1_ and *f*_2_ to one and estimating the latent covariance, all factor loadings, and all residual variances freely. We did not specify a mean structure. To recover the group difference, we provided max*LR* and max*LM* trees with a standard normally distributed informative covariate. The true cut point in the covariate point was central.

We explored the following three scenarios:

• *Testing measurement invariance:* We ignored heterogeneity in the latent covariance *ϕ* and tested only the (homogeneous) measurement part of the model by treating all factor loadings and residual variances as focus parameters. This scenario can be seen as an exploration of which covariates predict violations of strict measurement invariance.
• *Testing the latent covariance:* Only the (heterogeneous) latent covariance *ϕ* was treated as a focus parameter, and the remaining (homogeneous) parameters did not contribute to the assessment of potential splits. This scenario is akin to ignoring the covariates’ information on violations of measurement invariance and, instead, investigating differences on the latent level only.
• *No focus parameters:* No focus parameters were specified, and all parameters contributed to the evaluation of a potential split. This scenario served as a baseline.

Table 7 shows the percentage of max*LR* and max*LM* trees that split the sample and rejected the specific null hypothesis for a significance level of 5%. In the measurement invariance scenario, the SEM trees tested the null hypothesis that all parameters of the measurement model are homogeneous. Both max*LR* and max*LM* trees yielded error rates that were close to the optimal rate of 5%. In other words, the SEM tree methods successfully ignored the group difference in the covariance structure of the latent variables. Without focus parameters, the SEM trees tested the standard null hypothesis of complete parameter equivalency across groups. The max*LR* and the max*LM* trees rejected the null hypothesis in over 80% of the replications. If only the latent covariance *ϕ* was declared as a focus parameter, the power of both SEM trees to detect the group difference rose substantially and almost approached one. This finding highlights that the sensitivity of SEM trees for heterogeneity in a specific set of target parameters can be significantly enhanced by specifying focus parameters if differences with respect to the non-focus parameters can be safely ignored.

**TABLE 7:** Type I error and power to detect group differences.

Scenario	max*LR*	max*LM*
Testing measurement invariance	4.86	4.85
Testing the latent covariance	99.10	99.18
No focus parameters	82.97	81.21

*The first row shows the type I error rates and the second and third row the statistical power to detect group differences.*

### Simulation IV: Global Equality Constraints

Simulation IV aimed at investigating the utility of SEM trees with equality constraints and pointing out common pitfalls. Equality constraints are useful to incorporate prior knowledge about the homogeneity of specific parameters into a SEM tree. By constraining a parameter to equality, a so-called global constraint, this parameter is estimated once in the full sample, and the resulting estimate is used in all submodels. Constraining parameters increases a SEM tree’s sensitivity for group differences in the remaining parameters and might stabilize estimation. However, by erroneously constraining parameters to equality that are actually different in certain groups, a SEM tree can be severely misspecified.

We investigated the following conditions:

• *Group differences:* We tested two types of group differences. Either the latent covariance differed between groups (*ϕ*_*g*1_ = 0, *ϕ*_*g*2_ = 0.471) and the factor loading was homogeneous (*λ*_*g*1/*g*2_ = 0.837) or the latent covariance was homogeneous (*ϕ*_*g*1/g2_ = 0.471) and the factor loading differed (*λ_*g*_*_1_ = 0.837, *λ_*g*_*_2_ = 0.640). All other values were as shown in Figure 4. We generated 250 individuals per subgroup and provided the SEM trees with a standard normal covariate with a central cut point.
• *Equality constraints:* Either the heterogeneous parameter (the latent covariance *ϕ* or the factor loading *λ*), all factor loadings and residual variances of the factor *f*_2_, or no parameters were constrained to equality.

The empirical power of max*LR* and max*LM* trees for detecting heterogeneity for a significance level of 5% is shown in Table 8. Without equality constraints, both SEM tree methods showed a power of slightly above 80%. As expected, constraining a heterogeneous parameter reduced the power significantly, but the exact effect depended on the specific parameter. After constraining the heterogeneous latent covariance *ϕ*, there was no possibility for the trees to detect a difference between groups. The latent covariance *ϕ* was the only parameter associated with the correlation between the factors, and the group difference had no other parameter left to manifest. As a result, the power of max*LR* and max*LM* trees reduced to the type I error level. However, after constraining the heterogeneous factor loading *λ*, the trees still found significant group differences in 24.89% (max*LR*) and 22.65% (max*LM*) of the replications. This finding implies that the group difference in the factor loading *λ* were picked up by other unconstrained parameters of the measurement model. Finally, constraining the homogeneous parameters of the measurement model of factor *f*_2_ increased the power to detect differences in the latent covariance or the factor loading by roughly 10 percentage points.

**TABLE 8:** Power to detect group differences.

**Equality constraints**	**max*LR***	**max*LM***
***Group difference in the latent covariance ϕ***		
None	82.52	81.13
Latent covariance *ϕ*	5.71	5.52
Measurement model of *f*_2_	91.25	90.40
***Group difference in the factor loading* λ**		
None	83.20	81.44
Factor loading λ	24.89	22.65
Measurement model of *f*_2_	91.52	90.92

The results of Simulation IV suggest that constraining homogeneous parameters to equality can increase the power of SEM trees but also involves the risk of introducing severe misspecification. Constraining a heterogeneous parameter leads to a distorted picture of group differences as the SEM tree might or might not find significant heterogeneity in other parameters that are homogeneous across subgroups. Thus, it seems generally advisable to fully explore differences in all parameters or to use focus parameters rather than taking the risk of misspecifying trees by using inadequate equality constraints.

### Summary

In our simulation studies, the likelihood-ratio-guided *naïve* trees showed mixed results, confirming the known weaknesses of the approach. When provided with ordinal or dichotomous covariates, *naïve* trees showed an adequate control of type I errors and were among the best-performing methods in terms of power to detect heterogeneity and group recovery. However, with continuous covariates, *naïve* trees were overly conservative, resulting in too few type I errors and low power. The likelihood-ratio-guided *fair* trees showed overall the lowest power of all methods, resulting in a poor group recovery. In contrast to *naïve* trees, the type I error rate of *fair* trees was close to optimal, regardless of the measurement level of the provided covariates. Therefore, *fair* trees may be useful in very large samples where low power is less of an issue. The likelihood-ratio-guided max*LR* trees, that we implemented in the semtree package, resolved many of the weaknesses of the classical SEM tree methods *naïve* and *fair* and positioned themselves slightly above the score-guided max*LM* trees in terms of power, group recovery, and cut point precision. max*LR* trees and the score-guided max*LM* (for continuous covariates), and max*LM*_*O*_ trees (for ordinal covariates) exceeded other split selection approaches in conditions with group differences in multiple parameters associated with the random effects and non-central cut points. SEM trees guided by the score-based *DM* (for testing continuous covariates) and *WDM* (ordinal covariates) test statistics outperformed other methods in terms of power and group recovery when group differences were to be found in a single parameter describing the fixed slope. Different from *DM* trees, *WDM* trees were also sensitive to non-central cut points. Score-guided *CvM* trees performed better than other methods in detecting heterogeneity in the random effects when the cut point was central. Finally, the score-based *LM* trees for categorical covariates were slightly less powerful than the *naïve* method. Although max*LM*_*O*_ and *LM* trees were roughly on par with *naïve* trees, the score-based methods clearly outperformed *naïve* trees when provided with an additional continuous noise variable. Overall, all score-guided trees and the newly implemented max*LR* trees showed a satisfactory control of type I errors. The most striking difference between likelihood-ratio and score-guided SEM trees was the runtime. Whereas the runtime of all likelihood-ratio-based methods was excessive if one of the covariates under evaluation was continuous, score-guided trees were computed quickly. In summary, all newly implemented methods (max*LR* and the score-based methods) outperformed the original *naïve* and *fair* methods. Moreover, no single method under evaluation performed best across all situations, and all of the new methods had some unique advantages which may justify their use given certain conditions.

Regarding focus parameters and equality constraints, we found that both can successfully be applied to increase the power of SEM trees to detect group differences if there is either a clear set of target parameters or prior knowledge about homogeneous parameters available. Still, we discourage the use of equality constraints in favor of focus parameters, which allow exploring the effects of selected parameters without incurring misspecifications during the split evaluation.

## Discussion

In the present study, we introduced score-guided SEM trees as a fast and efficient way for growing SEM trees. Along with score-guided SEM trees, we also implemented a new likelihood-ratio-guided split selection based on the max*LR* statistic that solved many of the shortcomings of the original likelihood-ratio-guided SEM trees (Brandmaier et al., 2013b). We evaluated and compared the newly implemented and the original SEM tree approaches in a Monte Carlo simulation study. We investigated those cases in which users want to adjust the type I error rate for multiple testing of covariates. Overall, we conclude that the new split selection procedures are superior to the original split selection because they have higher statistical power and are unbiased in the selection of covariates that predict group differences in SEM parameters. Among the SEM tree methods, score-guided trees stand out due to their computational efficiency, making the use of SEM trees in large data sets feasible.

Our simulation studies evaluated three different likelihood-ratio-guided SEM tree approaches and five different score-guided SEM trees. The score-guided SEM trees were based on test statistics recently popularized in psychometrics by Merkle and Zeileis (2013) and Merkle et al. (2014) for studying heterogeneity in SEM parameters. When provided with continuous covariates, we found that guiding SEM trees with score-based tests significantly reduced the runtime of the trees. If solely provided with ordinal and categorical variables, score-guided SEM trees performed as well as likelihood-ratio-guided SEM trees. The large difference in the runtime of both approaches for continuous covariates can be attributed to the fact that the evaluation of a covariate by a likelihood-ratio-guided SEM tree requires the estimation of a MGSEM for every unique value of covariate. This leads to a large number of MGSEMs to be estimated as most values of continuous covariates are usually unique. However, score-guided SEM trees do not require the estimation of any MGSEMs for the evaluation of a covariate and, therefore, can be computed in little time.

Score-guided SEM trees and likelihood-ratio-guided trees based on the newly implemented max*LR* statistic also proved to be more powerful in detecting group differences than the original SEM tree methods if one of the covariates provided to the SEM tree were continuous. The low statistical power of the original SEM tree implementation is a side effect of a suboptimal correction of the selection bias. The low power of the *naïve* method for continuous covariates can be explained by the overcorrection of the Bonferroni adjusted *p*-values due to too many possible cut points in the continuous variables. The low power that the *fair* method displayed throughout all simulation conditions was because the *fair* selection method uses only half of the sample for selecting the best covariate.

Besides the evaluation of the original and newly proposed SEM tree methods, we also demonstrated the utility and pitfalls of trees with focus parameters and equality constraints. We showed that focus parameters are well suited to investigate specific hypotheses about parameter heterogeneity, such as different types of measurement invariance. Specifying equality constraints for homogeneous parameters increased the power of SEM trees for detecting group differences in the remaining parameters. We also demonstrated that misspecified equality constraints can obscure group differences and thus discourage this approach. As the effect of misspecified equality constraints can be hard to predict for a user, we recommend to explore differences in all parameters or to use focus parameters rather than risk to misspecify trees by using inadequate equality constraints.

The faster runtime of score-based tests is a major advantage for practical use and enables the wider adoption of SEM trees. The slow runtime of likelihood-ratio tests had made SEM trees unattractive if not impossible to run with large data sets on desktop computers. The runtime improvement may become even more important if one wishes to complement SEM tree inferences with resampling methods such as SEM forests (Brandmaier et al., 2016). SEM forests are a more robust alternative to single SEM trees if the overall importance of variables is of primary interest because small variations in the sample often lead to different trees. As SEM forests are based on hundreds if not thousands of trees, they will profit dramatically from the score-guided strategy.

The question remains which of the newly implemented methods should be used to estimate SEM trees. Our simulation results imply that all of the new methods have their unique strengths. However, in practice, when it is usually unknown how many of the model parameters are heterogeneous or if the subgroups are roughly equal in size, the advantages of the *DM*, *WDM*, and *CvM* statistics seem hard to exploit. Instead, the max*LR* (if computational feasible), max*LM*, max*LM*_*O*_, and *LM* trees statistics are best suited for situations without *a priori* knowledge about potential group differences. Moreover, if one is only interested in change in a specific parameter, specifying a focus parameter may represent an excellent alternative to the *DM* and *WDM* statistics.

Although SEM trees are a powerful and flexible method for investigating heterogeneity in SEMs, we want to stress that they are not always the most appropriate one. It is important to note that the performance of the SEM trees depends on the covariates available. If none of these covariates is in any way related to group differences, SEM trees will fail to detect any heterogeneity. In situations without informative covariates, researchers may resort to latent class or finite mixture models (Jedidi et al., 1997; Muthén and Shedden, 1999; Lubke and Muthén, 2005) for detecting heterogeneity. Latent class approaches automatically test for differences between all possible groups of individuals without requiring covariates. A disadvantage of these methods is that the number of subgroups needs to be pre-specified by the user. Another disadvantage of SEM trees is that they provide only sparse information about how a parameter changes with respect to a covariate. Recently, Arnold et al. (2019) suggested a framework called individual parameter contribution regression that allows modeling SEM parameter estimates as a linear function of covariates.

There are several limitations of our study. First, we focused narrowly on the semtree package for growing SEM trees and did not evaluate SEM trees estimated by the generic MOB algorithm from the partykit package. Ideally, a future study should aim to replicate our findings using MOB. Second, most of our simulations were performed using a linear latent growth curve model with only two types of group differences. Likely, different types of SEMs or parameter differences could have changed the performance of some of the methods under investigation. However, we would expect the general pattern of results to hold for other models as well. Third, for the sake of simplicity, we tested only a small number of uncorrelated covariates and did not test any covariate interactions. Fourth, we did not assess the influence of non-normally distributed data and model misspecification on the SEM trees. These remain topics for future research.

In summary, we found score-guided SEM trees to be fast, flexible, and powerful tools for investigating heterogeneity in SEM parameters. Based on our work, we suggest that score-guided split selection should become the new standard for estimating SEM trees and forests.

## Data Availability Statement

The simulated data presented in this study can be found in the following online repository: https://osf.io/k82y3/.

## Author Contributions

MA programmed the score-guided SEM tree implementation with the support of AB. MA designed and carried out the simulation study and wrote the manuscript with the support of MV and AB. MV revised the presentation of score-based tests and supervised the project. AB conceived the original idea and provided crucial code to link the score-guided SEM tree implementation to the existing semtree R package. All authors discussed the results and commented on the manuscript.

## Conflict of Interest

The authors declare that the research was conducted in the absence of any commercial or financial relationships that could be construed as a potential conflict of interest.

## Footnotes

1 https://github.com/brandmaier/semtree/commit/30ca7500e43ca99975dfe6b8917ef8f293beaeb3

2 https://osf.io/k82y3/
